# Parental Perceived Stress and Its Association With Childhood Obesity in Spain: The PASOS Study

**DOI:** 10.5888/pcd23.260028

**Published:** 2026-07-02

**Authors:** Silvia García, Marina Ródenas-Munar, Luis Cereijo, Marta Sevilla-Sanchez, Narcis Gusi, Santiago F. Gómez, Marcela González-Gross, Julia Wärnberg, Susana Aznar, Susana Pulgar, Lluís Serra-Majem, Adela Martín-Oliveros, Elena Marín-Cascales, Montse Fitó, Luis Carmona-Rosado, Miguel Ángel González-Valeiro, Jesús Sánchez Gomez, Paula Berruezo, Augusto G. Zapico, Isabel María Morales-Gil, Evelyn Martin-Moraleda, Estefanía Herrera-Ramos, Ana Mateos-Lardiés, Pedro E. Alcaraz, Helmut Schröder, Idoia Labayen, Cristina Bouzas, Josep A. Tur

**Affiliations:** 1 Research Group on Community Nutrition and Oxidative Stress, University of Balearic Islands-IUNICS, Palma de Mallorca, Spain; 2 Centro de Investigación Biomédica en Red Fisiopatología de la Obesidad y la Nutrición, Institute of Health Carlos III, Madrid, Spain; 3 Health Research Institute of Balearic Islands, Palma, Balearic Islands, Spain; 4 Public Health and Epidemiology Research Group, School of Medicine and Health Sciences, Universidad de Alcalá, Alcalá de Henares, Madrid, Spain; 5 Faculty of Sports Sciences and Physical Education, Universidade da Coruña, A Coruña, Spain; 6 Physical Activity and Quality of Life Research Group, Faculty of Sport Sciences, University of Extremadura, Cáceres, Spain; 7 Instituto de Investigación e Innovación en el Deporte, Cáceres, Spain; 8 Gasol Foundation Europe, Sant Boi de Llobregat, Barcelona, Spain; 9 CIBER de Epidemiología y Salud Pública, Instituto de Salud Carlos III, Madrid, Spain; 10 Cardiovascular Risk and Nutrition Research Group, Hospital del Mar Research Institute, Barcelona, Spain; 11 Nursing and Physiotherapy Department, University of Lleida, Lleida, Spain; 12 ImFINE Research Group, Department of Health and Human Performance, Universidad Politécnica de Madrid, Madrid, Spain; 13 EpiPHAAN Research Group, Faculty of Health Sciences, Universidad de Málaga, Instituto de Investigación Biomédica de Málaga, Málaga, Spain; 14 PAFS Research Group, Faculty of Sports Sciences, University of Castilla-La Mancha-Toledo Campus, Toledo, Spain; 15 Regional Unit of Sports Medicine of Principado de Asturias, Fundación Deportiva Municipal de Avilés, Avilés, Spain; 16 Research Institute of Biomedical and Health Sciences, University of Las Palmas de Gran Canaria, Las Palmas, Spain; 17 Preventive Medicine Service, Centro Hospitalario Universitario Insular Materno Infantil, Canarian Health Service, Las Palmas, Spain; 18 Sociedad Española de Farmacia Clínica, Familiar y Comunitaria, Spain; 19 UCAM Research Center for High Performance Sport, Universidad Católica de Murcia, Murcia, Spain; 20 Faculty of Sport, Universidad Católica de Murcia, Murcia, Spain; 21 ELIKOS Group, Institute for Sustainability and Food Chain Innovation, Department of Health Sciences, Public University of Navarre, Pamplona, Spain

## Abstract

**Introduction:**

Parental perceived stress (PPS) is proposed to be a factor influencing children’s weight status through behavioral and environmental mechanisms. We assessed the association between PPS and weight status among Spanish children and adolescents.

**Methods:**

We used data from the first wave of the PASOS (Physical Activity, Sedentarism, and Obesity in Spanish Youth) study (2019–2020). The study included 1,023 children and adolescents whose parents completed the Perceived Stress Scale. Anthropometric measurements and data on physical activity, sleep, screen time, and dietary quality were collected. We classified parents into “low” stress (<50th percentile on Perceived Stress Scale) and “high” stress (≥50th percentile on Perceived Stress Scale). We assessed the association between PPS and obesity, adjusted for parents’ perception of their own health and children’s general fitness. We conducted linear regression analysis between PPS and children’s body mass index (BMI) *z* score and waist-to-height ratio.

**Results:**

Children of parents with high PPS had higher mean BMI (19.9 [SD, 3.8] vs 19.5 [3.3]; *P* = .009) and poorer general fitness (*P* = .003) than children of parents with low PPS. The prevalence of obesity was higher among children of high-stress (vs low-stress) parents (4.0% vs 1.6%); this association remained significant after adjustment (adjusted OR = 2.76; 95% CI, 1.08–7.06; *P* = .03). The linear analysis showed positive associations between PPS and both children’s BMI *z* score and waist-to-height ratio.

**Conclusion:**

Higher PPS was associated with an increased likelihood of obesity in Spanish children and adolescents. These findings underscore the importance of addressing family stress in strategies for preventing childhood obesity.

**Summary**

**What is already known on this topic?**

Parental perceived stress (PPS) is a potential factor in the development of childhood obesity. Parents under stress may have less time, energy, or emotional resources to support the healthy eating and lifestyle routines of their children, thus promoting the development of childhood obesity.

**What is added by this report?**

In a Spanish national representative sample of children and adolescents, with parental data also collected, higher PPS was associated with higher likelihood of obesity, even after adjusting for significant children’s lifestyle variables.

**What are the implications for public health practice?**

This study highlights the importance of considering family stress and parental well-being in strategies to prevent childhood obesity and promote healthier lifestyle behaviors.

## Introduction

Childhood overweight and obesity are major public health challenges in Spain and worldwide, given their high prevalence and long-term health implications (1). It is crucial to identify not only traditional determinants, such as poor diet, physical inactivity, or excessive sedentary behavior, but also psychosocial factors that may influence obesogenic patterns within the family environment (2).

Parental perceived stress (PPS) is a potential factor in the development of childhood obesity (3). PPS is defined as the extent to which parents perceive their lives as unpredictable, uncontrollable, and overloaded, according to the conceptual framework of the Perceived Stress Scale (4). Parenting behaviors can be considered potential consequences of elevated stress rather than components of the definition itself. Parents experiencing high stress levels may have less time, energy, or emotional resources to support healthy routines, which could lead to less structured mealtimes, greater reliance on convenience foods, and reduced supervision of children’s screen time or physical activity (3). A systematic review concluded that different types of stress (general stress, parenting stress, or life events) may influence child weight outcomes through diverse behavioral and biologic pathways yet emphasized the need for further population-based studies controlling for mediators and confounders (5). From a physiologic perspective, chronic stress can disrupt hormonal balance, leading to higher cortisol levels and changes in appetite, sleep, and physical activity (6). Within the family setting, these stress-related mechanisms may promote an obesogenic environment, fostering unhealthy behaviors and weight gain in children. Indeed, evidence suggests that parental stress can indirectly contribute to child obesity through reduced meal planning, high consumption of energy-dense foods, and more permissive parenting styles (7,8).

To date, few studies in Spain have assessed the association between PPS and childhood obesity while also considering lifestyle behaviors such as physical fitness, sleep, screen time, and dietary quality as potential mediators or modifiers of this relationship. Although broader reviews highlight associations between parenting stress or parental behaviors and children’s lifestyle factors and body mass index (BMI), the role of PPS evaluated in conjunction with lifestyle mediators remains underexplored, particularly in Spanish population-based samples (3,9). This study aimed to assess the association between PPS and weight status among Spanish children and adolescents participating in the first wave of PASOS (Physical Activity, Sedentarism, and Obesity in Spanish Youth) study while accounting for key lifestyle-related factors.

## Methods

### Study design

The PASOS study was an observational, cross-sectional, multicenter, nationally representative study designed to assess physical activity, sedentary behaviors, and obesity-related indicators among Spanish children and adolescents (10).

### Participants, recruitment, data collection, and ethics

We used data from the first wave of the PASOS study (2019–2020), which included a representative sample of Spanish children and adolescents (hereinafter referred to only as children) and their parents or legal guardians (hereinafter referred to only as parents). Schools were invited to participate, and those agreeing to follow the study protocol were included. Participants were recruited through 247 educational centers proportionally distributed among 17 autonomous communities across Spain to ensure national representativeness. The study was conducted from March 2019 through February 2020 and included boys and girls aged 8 to 16 years. Additional inclusion criteria were considered, such as attendance at the participating school, signed consent, and assent from participants aged 12 years or older. Written informed consent was obtained from both parents and participating children. The study enrolled 3,802 children. Data collection was conducted by trained researchers belonging to 13 collaborating research groups and more than 70 field staff members. Children with intellectual disabilities that impeded questionnaire completion were excluded from participation; such exclusions were confirmed jointly by teachers and parents before final enrollment. Of the 3,802 enrolled children, we excluded 2,779 for not having available data on parental stress levels; the final sample consisted of 1,023 participants.

The study complied with the ethical standards of the Declaration of Helsinki, was approved by the Ethics Committee of the Fundació Sant Joan de Déu, and was registered in the International Standard Randomized Controlled Trial Number registry (ID 34251612) (11). Details on the study protocol are available elsewhere (10).

To mitigate the potential for variance between observers, field researchers participated in a 1-day training session conducted by the Gasol Foundation. The study collected data from 2 sources: validated questionnaires and anthropometric measurements.

Validated questionnaires were administered to children to collect data on lifestyle variables and to parents to collect data on lifestyle variables, socioeconomic factors, and environmental variables. For this analysis, we extracted data on the following: parent’s age, child’s sex (male, female), parent’s perception of own health (excellent, very good, good, poor or bad), and parent’s employment status (working, home working, unemployed, student, or other [retirement or permanent disability]).

Anthropometric measurements included weight, height, and waist circumference of the participating children.

### Parental perceived stress

PPS was assessed by using the Perceived Stress Scale (4), which was completed online by parents whose children participated in the study. The Perceived Stress Scale measures the degree to which personal life situations are considered stressful; it consists of 14 questions asking how often a person felt or thought in a certain way during the previous month. Each question has 5 possible answers from 0 (never) to 4 (very often). Seven positive items (questions 4, 5, 6, 7, 9, 10 13) are reversed scored (ie, assigned values such that 0 = 4, 1 = 3, 2 = 2, 3 = 1, 4 = 0). The scores of all 14 items are then summed. Scores can range from 0 to 56, with higher scores indicating a higher degree of stress.

PPS was reported by the caregiver identified as “Adult 1,” defined as the parent who spent the most time caring for the child. Only this caregiver completed the questionnaire, and only their data were used for the analysis; therefore, no multiple responses were obtained for the same participant.

### Physical fitness, sleep duration, sedentary behavior, and diet of the children

Perceived physical fitness was assessed by using the validated International Fitness Scale questionnaire, which shows good reliability (coefficients ranging from 0.68 to 0.80) (12). Sleep duration on weekdays and weekends was calculated as the difference between bedtime and wake-up time by using the validated Sleep Habits Survey (13); participants were classified according to compliance with the National Sleep Foundation sleep recommendations (14). Screen time was assessed by using the validated the Healthy Lifestyle in Europe by Nutrition in Adolescence (HELENA) screen time–based sedentary behavior questionnaire (κ > 0.7) (15), which considered total daily screen time on weekdays and weekends and adherence to the American Academy of Pediatrics’ recommendation of less than 120 minutes per day (16). Dietary quality was evaluated by using the 16-item KIDMED index, a validated instrument for assessing adherence to the Mediterranean diet among Spanish children and adolescents (17–19), with total scores ranging from −4 to 12 and categorized as low, medium, or high adherence. All questionnaires were self-completed by children.

### Anthropometric measurements

Measurements of body weight (kg), height (cm), and waist circumference (cm) of the participating children were performed following the standard protocol established by the World Health Organization (20). For body weight measurement, a SECA 899 electronic scale (SECA GmbH) was used; for height, a SECA 217 portable stadiometer (SECA GmbH) was used; and for waist circumference, a SECA 201 flexible tape measure (SECA GmbH) was used. Two waist circumference measurements were taken, and if the difference between the 2 measurements exceeded 1 cm, a third measurement was taken. From these measurements, BMI was calculated as weight in kilograms divided by height in meters squared. Weight status was based on the Orbegozo growth reference categories, which uses age- and sex-specific BMI cutoff points derived from Spanish percentile tables (21). The standardized BMI z score was compared with the WHO 2007 growth standards for children and adolescents (22), according to the BMI *z* score age and sex cutoffs (overweight and obese >1 SD; normal weight ≥−2 SD and ≤1 SD; and underweight <−2 SD). A waist-to-height ratio was computed by dividing waist circumference by height to provide a continuous marker of adiposity (23).

### Statistical analysis

For statistical analysis, we used SPSS version 27.0 (IBM SPSS Statistics for Windows). We used Perceived Stress Scale scores to obtain the grouping variable. Scores were divided into 2 groups: we assigned “low perceived stress” to parents whose Perceived Stress Scale score was at or below the 50th percentile and “high perceived stress” to parents who were above the 50th percentile. We tabulated descriptive statistics for quantitative variables as mean (SD). We used the Student *t* test to assess differences between groups. For categorical variables, we tabulated frequency and percentage. We used χ^2^ analysis to examine between-group differences in categorical variables. Statistical significance was determined at *P* < .05.

We used binary logistic regression to assess the association between the grouping variable (low or high perceived stress) and weight status–related variables. We calculated odds ratios (ORs) and their 95% CIs, with low perceived stress used as the reference category. Continuous anthropometric variables (weight, height, BMI, and waist circumference) were categorized by using the sample median as the cutoff, while weight status and abdominal obesity were analyzed as dichotomous variables (yes/no). The OR was calculated twice: first, unadjusted (crude OR), and second, adjusted for parent’s perception of own health, physical fitness of the child, as well as child’s sex and age, as key covariates. Finally, we performed a linear regression analysis to examine the association between total Perceived Stress Scale and children’s BMI *z* score and waist-to-height ratio.

## Results

### Sociodemographic characteristics of children and parents

We observed no differences in the mean age of children or parents between the low-stress and high-stress groups (Table 1). However, children with parents in the high-stress group had a slightly higher mean BMI than children with parents in the low-stress group (19.9 [SD, 3.8] vs 19.5 [SD, 3.3]; *P* = .009). The distribution of sex and the prevalence of abdominal obesity did not differ between groups. We found a higher proportion of children with obesity among parents in the high-stress group (4.0%) than among parents in the low-stress group (1.6%), but this difference was not significant (*P* = .08).

**Table 1:** Sociodemographic Characteristics of Children, Adolescents, and Parents According to Parental Perceived Stress,^a^ Spain, 2019–2020^b^

Characteristic	Parent’s Perceived Stress Scale^c^		*P* value^d^
	Low (n = 549)	High (n = 474)	
**Child, mean (SD)**			
Age, y	12.4 (2.3)	12.3 (2.4)	.10
BMI, kg/m^2^	19.5 (3.3)	19.9 (3.8)	.009
**Parent, mean (SD)**			
Age, y	45.1 (5.5)	44.8 (5.8)	.63
BMI, kg/m^2^	24.9 (4.1)	25.3 (4.5)	.12
**Child’s sex, no (%)**			
Female	277 (50.5)	231 (48.7)	.58
Male	272 (49.5)	243 (51.3)	
**Child’s weight status (both sexes), no. (%)**			
Undernutrition	8 (1.5)	5 (1.1)	.08
Underweight	33 (6.0)	35 (7.4)	
Healthy weight	414 (75.4)	330 (69.6)	
Overweight	85 (15.5)	85 (17.9)	
Obesity	9 (1.6)	19 (4.0)	
Abdominal obesity (waist-to-height ratio >0.5)	93 (16.9)	92 (19.4)	.32
**Parent’s perception of own health, no. (%)**			
Excellent	42 (7.7)	19 (4.0)	<.001
Very good	216 (39.3)	92 (19.4)	
Good	272 (49.6)	306 (64.5)	
Poor	16 (2.9)	53 (11.2)	
Bad	3 (0.5)	4 (0.8)	
**Parent’s employment status, no (%)**			
Working	420 (76.5)	342 (72.2)	.10
Home working	66 (12.0)	66 (13.9)	
Unemployed	31 (5.6)	40 (8.4)	
Student	1 (0.2)	4 (0.8)	
Other (retirement or permanent disability)	31 (5.6)	22 (4.6)	

Abbreviation: BMI, body mass index.

a Defined as the extent to which parents perceive their lives as unpredictable, uncontrollable, and overloaded, according to the conceptual framework of the Perceived Stress Scale (4).

b Data source: First wave of the PASOS (Physical Activity, Sedentarism, and Obesity in Spanish Youth) Study (10).

c Perceived Stress Scale scores were divided into 2 groups: “low perceived stress” to parents whose score was at or below the 50th percentile and “high perceived stress” to parents who were above the 50th percentile.

d Differences in groups were examined by using χ^2^ and Student *t* test.

Parental health perception differed significantly between stress groups (*P* < .001). Parents with high perceived stress were less likely to report their health as excellent or very good (4.0% and 19.4%, respectively) and more likely to rate it as good or poor (64.5% and 11.2%, respectively) compared with parents with low perceived stress. Employment status did not differ significantly between groups.

### Children’s physical fitness, sleep duration, screen time, and diet quality

Children whose parents reported high stress had poorer general fitness than children whose parents reported low stress, with a higher proportion classified as having bad to acceptable fitness levels (30.6% vs 22.8%; *P* = .003) (Table 2). Similarly, cardiorespiratory fitness was significantly lower among children whose parents reported high stress (*P* = .02). We observed no differences between groups for muscle strength, speed and agility, or flexibility. The proportion of children who met the daily recommendation for moderate-to-vigorous physical activity was significantly lower among children whose parents reported high stress (77.8%) than among children whose parents reported low stress (82.7%) (*P* = .005). We found no differences between stress groups on weekdays or weekends.

**Table 2:** Children’s and Adolescents’ Physical Fitness, Sleep Duration, Screen Time, and Diet Quality According to Parental Perceived Stress,^a^ Spain, 2019–2020^b^

Characteristic	Parent’s Perceived Stress Scale^c^		*P* value^d^
	Low (n = 549)	High (n = 474)	
**Physical fitness, no. (%)**			
General fitness status			
Bad to acceptable	125 (22.8)	145 (30.6)	.003
Good to very good	424 (77.2)	329 (69.4)	
Cardiorespiratory fitness			
Bad to acceptable	152 (27.7)	145 (30.6)	.02
Good to very good	397 (72.3)	329 (69.4)	
Muscle strength			
Bad to acceptable	190 (34.6)	161 (34.0)	.20
Good to very good	359 (65.4)	313 (66.0)	
Speed and agility			
Bad to acceptable	175 (31.9)	182 (38.4)	.26
Good to very good	374 (68.1)	292 (61.6)	
Flexibility			
Bad to acceptable	315 (57.4)	168 (35.4)	.89
Good to very good	234 (42.6)	306 (64.6)	
Moderate-to-vigorous physical activity recommendation achieved	454 (82.7)	369 (77.8)	.005
**Sleep duration**			
Total sleep time, mean (SD), hours per weekday	9.0 (1.3)	8.9 (1.3)	.16
Total sleep time, mean (SD), hours per weekend	9.8 (1.6)	9.9 (1.6)	.62
Sleep time recommended achieved on weekdays, no. (%)	341 (62.1)	296 (62.4)	.96
Sleep time recommended achieved on weekends, no. (%)	276 (50.3)	242 (51.1)	.84
**Screen time**			
Total screen time, mean (SD), minutes per weekday	155.9 (136.8)	168.9 (144.3)	.06
Total screen time, mean (SD), minutes per weekend	265.7 (166.5)	263.7 (172.7)	.13
Screen time recommendation achieved on weekdays, no. (%)	272 (49.5)	216 (45.6)	.13
Screen time recommendation achieved on weekends, no (%)	114 (20.8)	121 (25.5)	.09
**Diet quality^e^**			
KIDMED score, mean (SD)	7.18 (2.4)	6.81 (2.5)	.99
KIDMED adherence			
Low	40 (7.3)	42 (8.9)	.07
Medium	262 (47.8)	249 (52.5)	
High	247 (45.0)	183 (38.6)	

a Defined as the extent to which parents perceive their lives as unpredictable, uncontrollable, and overloaded, according to the conceptual framework of the Perceived Stress Scale (4).

b Data source: First wave of the PASOS (Physical Activity, Sedentarism, and Obesity in Spanish Youth) Study (10).

c Perceived Stress Scale scores were divided into 2 groups: “low perceived stress” to parents whose score was at or below the 50th percentile and “high perceived stress” to parents who were above the 50th percentile.

d Differences in groups were examined by using χ^2^ and Student *t* test.

e Evaluated by using the 16-item KIDMED index, a validated instrument for assessing adherence to the Mediterranean diet among Spanish children and adolescents (17–19), with total scores ranging from −4 to 12 and categorized as low, medium, or high adherence.

Mean screen time tended to be higher among children whose parents reported high stress, both on weekdays (168.9 [SD, 144.3] vs 155.9 [136.8] min/d; *P* = .06) and weekends (263.7 [SD, 172.7] vs 265.7 [SD, 166.5] min/d, *P* = .13), but these differences were not significant. We found no differences in KIDMED scores between children whose parents reported low stress and children whose parents reported high stress (7.18 [SD, 2.4] vs 6.81 [SD, 2.5]; *P* = .99). A higher proportion of children whose parents reported high stress had low adherence to the Mediterranean diet compared with children whose parents reported low stress (8.9% vs 7.3%). Medium adherence was slightly more common among children whose parents reported high stress (52.5% vs 47.8%), while high adherence was more frequent among children whose parents reported low stress (45.0% vs 38.6%), but these differences were not significant (*P* = .07).

### Anthropometric measurements

We observed no associations between parental stress groups and children’s weight, height, BMI, or waist circumference, either in the crude or adjusted models (Table 3). We found no differences between parental stress groups for undernutrition, underweight, healthy weight, or overweight. Children whose parents reported high stress had a significantly higher likelihood of obesity compared with those whose parents reported low stress in both crude (OR = 2.51; 95% CI, 1.12–5.59; *P* = .02) and adjusted (OR = 2.76; 95% CI, 1.08–7.06; *P* = .03) models.

**Table 3:** Association of Children’s and Adolescents’ Anthropometric Measurements According to Parental Perceived Stress,^a^ Spain, 2019–2020^b^

Measurement	Perceived Stress Scale^c^		*P* value^d^
	Low (n = 528)	High (n = 544)	
**Weight in kg^e^**			
Crude OR	1 [Reference]	1.05 (0.82–1.35)	.70
Adjusted OR^f^	1 [Reference]	0.95 (0.72–1.27)	.74
**Height in m^e^**			
Crude OR	1 [Reference]	1.05 (0.82–1.34)	.73
Adjusted OR^f^	1 [Reference]	1.05 (0.79–1.38)	.76
**BMI^e^**			
Crude OR	1 [Reference]	1.20 (0.93–1.54)	.16
Adjusted OR^f^	1 [Reference]	1.05 (0.79–1.40)	.75
**Waist circumference in cm^e^**			
Crude OR	1 [Reference]	1.20 (0.94–1.54)	.15
Adjusted OR^f^	1 [Reference]	1.05 (0.79–1.40)	.74
Weight status^g^			
**Undernutrition**			
Crude OR	1 [Reference]	0.72 (0.23–2.22)	.57
Adjusted OR^f^	1 [Reference]	0.82 (0.25–2.71)	.75
**Underweight**			
Crude OR	1 [Reference]	1.25 (0.76–2.04)	.38
Adjusted OR^f^	1 [Reference]	1.17 (0.68–2.01)	.56
**Healthy weight**			
Crude OR	1 [Reference]	0.76 (0.58–1.00)	.05
Adjusted OR^f^	1 [Reference]	0.83 (0.61–1.14)	.26
**Overweight**			
Crude OR	1 [Reference]	1.19 (0.86–1.66)	.29
Adjusted OR^f^	1 [Reference]	1.03 (0.71–1.51)	.86
**Obesity**			
Crude OR	1 [Reference]	2.51 (1.12–5.59)	.02
Adjusted OR^f^	1 [Reference]	2.76 (1.08–7.06)	.03
**Abdominal obesity^g^**			
Crude OR	1 [Reference]	1.18 (0.86–1.62)	.32
Adjusted OR^f^	1 [Reference]	0.98 (0.68–1.42)	.92

Abbreviations: BMI, body mass index; OR, odds ratio.

a Defined as the extent to which parents perceive their lives as unpredictable, uncontrollable, and overloaded, according to the conceptual framework of the Perceived Stress Scale (4).

b Data source: First wave of the PASOS (Physical Activity, Sedentarism, and Obesity in Spanish Youth) Study (10).

c Perceived Stress Scale scores were divided into 2 groups: “low perceived stress” to parents whose score was at or below the 50th percentile and “high perceived stress” to parents who were above the 50th percentile.

d *P* < .05 considered significant.

e Continuous anthropometric measurements were categorized by using the sample median as the cutoff.

f Adjusted by parent’s perception of own health and general fitness status, age, and sex of the child.

g Weight status and abdominal obesity were analyzed as dichotomous variables (yes/no).

### Linear regression analysis

Higher PPS was positively associated with both children’s BMI *z* score and waist-to-height ratio (Table 4). PPS showed a small but significant effect on BMI z score (*P* = .03) and waist-to-height ratio (*P* = .03). Although the effect sizes were modest (*R* ^2^ = 0.004 for BMI *z* score and 0.005 for waist-to-height ratio), the direction of the associations indicated that higher PPS was related to slightly higher adiposity indicators in children.

**Table 4:** Linear Regression Analysis Between Parental Perceived Stress^a^ and Children’s BMI *z* Score and Waist-to-Height Ratio, Spain, 2019–2022^b^

Value	BMI *z* score	Waist-to-height ratio
*R* (correlation coefficient)	0.067	0.068
*R* ^2^ (determination coefficient)	0.004	0.005
*y =*	0.23 + (0.01 × *x*)	0.44 + [(4.85 × 10^−4^) × *x*]
95% CI	0.001 to 0.019	0.000048 to 0.001
*P* value	.03	.03

Abbreviation: BMI, body mass index.

a Defined as the extent to which parents perceive their lives as unpredictable, uncontrollable, and overloaded, according to the conceptual framework of the Perceived Stress Scale (4). Scores were divided into 2 groups: “low perceived stress” to parents whose score was at or below the 50th percentile and “high perceived stress” to parents who were above the 50th percentile.

b Data source: First wave of the PASOS (Physical Activity, Sedentarism, and Obesity in Spanish Youth) Study (10).

## Discussion

We found that children whose parents reported higher PPS levels had a higher likelihood of obesity, even after adjusting for potential confounders such as parental health perception and children’s general physical fitness. We observed no associations for overweight status or other anthropometric measurements. These results suggest that PPS may play a relevant role in the development or maintenance of obesity in children, rather than influencing milder degrees of excess weight. Moreover, we conducted linear regression models using continuous anthropometric indicators (BMI z score and waist-to-height ratio) to increase statistical power. These analyses supported the main findings, showing that higher PPS was positively associated with slightly higher BMI z score and waist-to-height ratio values. Although the effect sizes were small, these continuous associations reinforce the robustness of the relationship between PPS and children’s adiposity outcomes.

Previous studies showed that PPS is associated with less healthy dietary patterns in children and a high risk of overweight or obesity (5,24,25). High PPS was consistently linked to high BMI and obesity-related behaviors among children, particularly increased fast-food consumption (24–26). These findings are in line with our study results, where high PPS was also associated with high BMI among children.

In our study, higher PPS was also associated with worse general fitness status. Some evidence suggests that PPS may reduce children’s physical activity, although this relationship is not yet clearly established (25,27,28). For example, many parents report that the high cost of organized sports and activity is a reason for stress, which may limit their ability to enroll their children in physical activity programs and thus indirectly reduce children’s activity levels (29). Conversely, one study (25) found that higher PPS was associated not only with higher child BMI and fast-food consumption but also with increased physical activity, suggesting that stress may not always suppress movement in children. In a recent study, higher daily PPS was directly associated with lower child activity on the same day, although lagged effects showed a potential compensatory increase in activity the next day, especially among girls (28).

One factor that may also mediate the association between PPS and childhood obesity is the way that parents adapt their feeding practices during stressful periods. A clear example is the COVID-19 pandemic, during which both general and parenting stress increased substantially. Higher levels of stress were linked to the use of food to regulate children’s emotions and behaviors, and to parents’ perceptions of problematic eating patterns in their children (30). During the pandemic, PPS was also related to higher consumption of sweet and savory snacks mediated by snack parenting practices (31). Aside from the COVID-19 context, unhealthy eating habits have also been related to PPS and children’s behavioral problems (32). Similarly to our study, no relation between PPS and dietary quality has been found in other PPS related studies (33). We observed no differences in adherence to a healthy diet, such as the Mediterranean diet, between children with parents reporting low stress and children with parents reporting high stress. Overall, although the evidence to date remains somewhat inconsistent, parental psychologic stress appears to influence the home food environment and may represent an important intervention tactic for improving children’s dietary patterns. However, this association is likely to be complex and may be mediated by additional factors beyond diet, which could also contribute to the increased risk of obesity observed in children exposed to higher levels of parental stress.

Our findings suggest that lifestyle behaviors such as physical activity, dietary habits, and family routines may act as intermediate pathways linking parental stress and childhood obesity. However, given the cross-sectional design of our study, the temporal sequence between these factors cannot be established, and therefore mediation pathways should be interpreted with caution and further explored in longitudinal research. Additionally, we used descriptive analyses of lifestyle behaviors to inform the selection of covariates in the adjusted models. Variables showing relevant differences according to parental stress, such as physical activity, were considered in the OR analyses to reduce potential confounding.

Our study examined data on children’s sleep duration and screen time, and we found no differences between PPS groups. Previous research reported inconsistent findings (27,34–36). Some research showed that when parents feel more stressed, their children may end up using screens for longer (37), possibly because stressed caregivers have a decreased ability to supervise or set limits (36). Other studies reported that high stress can have the opposite effect, prompting some parents to apply household rules more firmly, which can reduce the amount of time children spend on screens (35). These contrasting results may help to explain why we observed no differences in our sample.

Previous evidence on sleep time showed a direct relationship between screen exposure and sleep duration, with higher screen use generally associated with shorter sleep in children and adolescents (34). Other studies suggested that PPS may arise as a consequence of children’s sleep difficulties rather than the reverse (38). Furthermore, research has consistently reported strong associations between PPS and children’s sleep problems as well as links between parental mental health and children’s sleep disturbances (39). Together, these findings highlight the complex and bidirectional relationships between children’s sleep patterns, screen habits, and family stress.

Although the literature shows inconsistent associations between parental stress and children’s physical activity, screen time, sleep duration, or overall diet quality, evidence consistently links higher parental stress with higher child BMI and increased fast-food consumption. These findings align with our study results and highlight fast-food intake as a relevant obesity-related behavioral factor that warrants further examination.

Finally, our findings also suggest that parents’ perception of their own health may be linked to parental stress, offering an additional pathway through which stress could influence childhood obesity. Parents who view their own health as poorer often report higher stress levels, which may limit their ability to maintain consistent, health-promoting routines at home and may influence children’s obesity risk indirectly (3,40,41). Overall, these results highlight that parents’ health and psychosocial well-being form part of a broader context that may shape children’s lifestyle patterns and weight outcomes. Taken together, these findings emphasize the need to consider parental psychosocial factors when addressing childhood obesity because they may shape children’s health trajectories.

### Strengths and limitations

The main strength of this study was the use of a representative sample of Spanish children, together with their parents. Although we used data from an analytic subsample (n = 1,023) of the larger PASOS study (n = 3,802), our study provides valuable insights into the association between PPS and childhood obesity. The standardized procedures used for anthropometric measurements and physical fitness assessments also enhance the reliability of the data. The inclusion of multiple lifestyle-related variables, such as physical activity, sleep duration, screen time, and dietary quality, provides a broader understanding of the context in which parental stress may influence child health. Adjusted OR models, stratified by parents’ perception of their own health, general fitness status of the child, and child’s sex and age, allow consideration of possible confounders in the relationship between parental stress and child obesity risk. The inclusion of linear regression analyses using continuous adiposity indicators (BMI *z* score and waist-to-height ratio) is an additional strength, as these models provide greater statistical power compared with categorical approaches. However, our study has several limitations. First, the cross-sectional design precludes establishing causal relationships between PPS and children’s weight status. Second, key variables such as PPS, perceived health, dietary patterns, sleep duration, and screen time were assessed through self-reported questionnaires, which may be subject to recall or social desirability bias. In addition, the potential for selection bias related to participation and missing data, as well as residual confounding despite statistical adjustment, may still exist. The PASOS study was designed to be nationally representative; our analytic subsample may slightly limit the generalizability of the findings to the broader population of children in Spain and must be considered when interpreting the results. Finally, it cannot extrapolate to other populations.

### Conclusion

In this nationally representative sample of Spanish children, with parental data also collected, higher PPS was associated with a higher likelihood of obesity, even after adjusting for significant children lifestyle variables, such as physical activity, and parents’ perception of their own health. We found no differences between children in overweight status or other anthropometric indicators according to PPS, suggesting that PPS may be more strongly linked to more severe excess weight. However, linear regression analyses using continuous adiposity markers revealed small but positive associations with PPS, which highlights the importance of considering family stress and parental well-being in strategies aimed at preventing childhood obesity and promoting healthier lifestyle behaviors.
